# The Correlation Between Age and Bleeding Volume in Haemorrhagic Stroke Using Multi Slice CT at District Hospitals in Jakarta

**DOI:** 10.5539/gjhs.v8n4p152

**Published:** 2015-08-06

**Authors:** Tatan Saefudin, Nursama Heru Apriantoro, Ekaputra Syarif Hidayat, Schandra Purnamawati

**Affiliations:** 1 Department of Radiodiagnostic and Radiotherapy, Politeknik Kesehatan Jakarta II 12120, Indonesia; 2 Faculty of Medicine, University of Jenderal Soedirman, Purwokerto 53112, Indonesia

**Keywords:** bleeding volume, haemorrhagic stroke, multi slice computed tomography

## Abstract

Haemorrhagic Stroke is a common disease in Indonesia. The best imaging modality for this disease is Multi Slice Computed Tomography Scanning (MSCT), as it may help strengthening the diagnosis as well as determining the brain bleeding volume. This study aimed to show correlation between bleeding volume of the brain and patient’s age using cross-sectional approach. The 68 samples in this study were taken from secondary data from Head CT Scan of Haemorrhagic Stroke cases. Brain bleeding volume is the dependent variable, obtained through slice thickness of 5 mm and ABC/2 method with software measurement in MSCT Scan device. The independent variable of this study is the patient’s age. The result of the study was the average brain’s bleeding volume of 21.76 ml ± 2.48 ml (range of 1.04 ml to 94.73 ml). The slice thickness using ABC/2 method, has a significant correlation with brain’s bleeding volume in MSCT Scan examination, with correlation coefficient value r of 0.79. Brain bleeding volume in patients who have ages lower than 50 years and more or equal to 50 years were (18.93 ± 3.26) ml and (23.53 ± 3.47) ml respectively. There is no correlation between age and brain’s bleeding volume in haemorrhagic stroke cases, with p value of 0.18, r = 0.19.

## 1. Introduction

Stroke is the second leading cause of death worldwide, and one of the leading causes of disability (O’Donnell & Yusuf, 2009; Ikram et al., 2012; Lozano et al., 2012). Intra cerebral haemorrhage is the second most common subtype of stroke after ischemic stroke and accounts for approximately 10% to 20% of all strokes (Feigin, 2009).

The prevalence of stroke in Caucasian population ranges between 500-600 per 100,000 people in New Zealand, 1445 per 100,000 people in France, 620 per 100,000 people in China, 690 per 100,000 people in Thailand. The prevalence in developing countries also varies (WHO, 2004).

Stroke is a leading cause of death and disability affecting about 16 million first-ever strokes occur in the world, causing a total of 5.7 million deaths every year (Strong et al., 2007). David reported each year in USA 500,000 people had stroke and 150,000 died. The overall prevalence is 750 per 100,000 (Davis, 2001). Prediction without intervention, the number of deaths from stroke will rise to 6.3 million in 2015 and 7.8 million by 2030 with largely in poor countries (Strong et al., 2007; Pandian et al., 2007).

In Indonesia, the main causes of death at all ages are: stroke (15.4%), injuries (6.5%), according to basic health survey (Ministry of Health of Indonesia, 2007). In 2013, stroke is the leading cause of death in almost all hospitals in Indonesia, around 15.4 %. There has been increasing of 8.3 per 1000 people in 2007 to 12.1 per 1000 people in 2013. The highest stroke prevalence of the disease is in North Sulawesi of 10.8 per 1000 people, Yogyakarta of 10.3 per 1000 people, Bangka Belitung of 9.7 per 1000 people and Jakarta as capital city of 9.7 per 1000 people (Ministry of Health, 2014).

Much of the epidemiologic research on intra cerebral haemorrhage has concentrated on identifying risk factors for intra cerebral haemorrhage, such as hypertension (O’Donnell, 2010), smoking habit (Zhang et al., 2011), alcohol consumption (O’Donnell, 2010; Zhang et al., 2011; Wieberdink, 2011), medical history of hypercholesterolemia and hyperlipidaemia (Wieberdink, 2011), aging process (Lindsay & Bone, 2004).

The most important modality to examine and diagnose stroke is Brain Computerized Tomography (CT). CT scan is a radiography examination technique, producing object images as axial and transversal body slices through tomography principles, completed with computerized system as data processing device (Bushong, 2008; Seeram, 2013). In CT process, the examination will take 15-20 minutes. It is excellent to detect intracranial bleeding (Hoggard, 2002). This device is capable of measuring the width of bleeding in haemorrhagic stroke patient’s brain.

Correct measurement of brain bleeding volume is essential to determine proper medical action and treatments. Bleeding volume can be measured using computer software in MSCT Scan device, to reveal an accurate brain bleeding volume in stroke patients through mathematical algorithm. Inaccurate bleeding volume measurement in haemorrhagic stroke patient will cause miss calculation of bleeding volume which may lead to inappropriate medical action and treatment, affecting the result of curative and disability limitation for stroke patient. In other word, accurate bleeding volume measurement in haemorrhagic stroke cases will help determining proper management to increase the chance of patient’s survival as well as patient’s quality of life.

The value of manual bleeding volume measurement is subjective to each radiologist expertise, as they had to calculate the volume of an irregular shape. The manual brain bleeding volume is different than computerized calculation. It may affect the decision making of medical actions and treatment for haemorrhagic stroke patient. Using the available software in CT scan multi slice device, brain bleeding volume can be accurately measured. The aim of this study is to discover the result of brain bleeding volume measurement and it’s affecting factors in haemorrhagic stroke patient using multi slice CT scan modality.

## 2. Methods

The study was done at Koja and Cengkareng Hospital in Jakarta Indonesia using Siemens Somatom Emotion 6 and Philips Brilliance 6 respectively. The examination procedure; patient supine position with head first to the gantry, blanket, head rest and head strap for comfort. Using following parameters; 5 mm slice thickness from the inferior orbital margin to the vertex of skull, window cerebrum in HU, 512x512 matrix size, 130 kV, 270 mAs, Field of View (FOV) for Brain and and Multiplanar Reconstruction CT Program (CT/MPR). The 68 samples were taken from secondary data of haemorrhagic stroke Head CT-scan examination. Brain haemorrhage volume was obtained using ABC/2 method, as reported by Kothari *et al* (1996). According to the data, the slice thickness was 5 mm. Statistical design of this study was analytic-quantitative with cross-sectional approach method. Data collection and analysis of this study used descriptive single-variable analysis. Bi-variable analysis aimed to reveal the correlation between independent variable, which is patient’s age and brain bleeding volume as dependent variable. The process of bivariate analysis was done using statistical software aid with correlation analysis, linier regression, and *t* test. Brain bleeding volumes were classified into four categories; small, moderate, large, and very large.

## 3. Results

**Figure 1:**
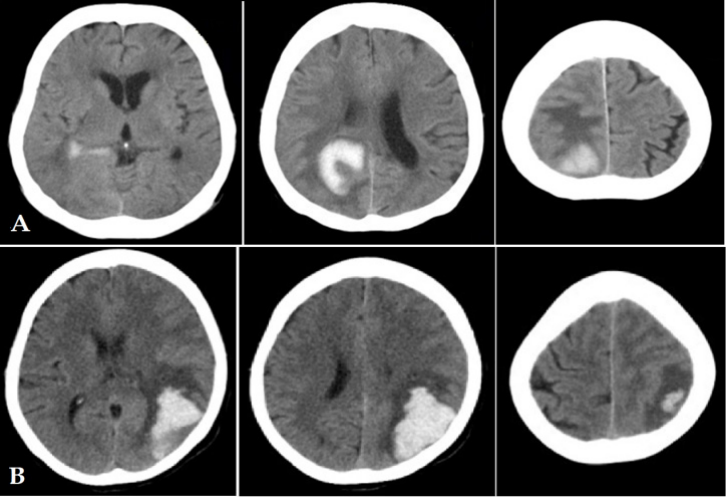
Head MSCT Imaging of Haemorrhagic Stroke patients in A) Koja Hospital B) Cengkareng Hospital

The statistical description of haemorrhagic volume in 68 patients was shown in Table 1. The mean value is (21.76 ± 2.48) ml, ranged from 1.94 ml to 94.73 ml. The calculation result of brain bleeding volume was obtained from slice thickness of selected patient which appear on the monitor screen. Evaluation process for bleeding area was done using ABC/2 method. Figure 1 shows samples of Head MSCT scan imaging with haemorrhagic stroke from Koja and Cengkareng hospitals. The correlation between number of slice and brain bleeding volume variables was shown in Table 2. It has a strong correlation in positive direction, the *R value* is 0.79. According to Kendall’s tau correlation non parametric test, *p* value is 0.00. This shows that the slice thickness using ABC/2 method is an accurate method to calculate brain bleeding volume in the study.

**Table 1:** Statistical value of bleeding volume (in ml)

Descriptive	Statistic Value	Std Error
N samples	68.00	
Mean	21.76	2.48
95% confidence interval of Mean		
Lower Bound	16.82	
Upper Bound	26.71	
Median	15.97	
Variance	417.36	
Std Deviation	20.43	
Minimum	1.04	
Maximum	94.73	
Skew	1.83	0.29
Kurtosis	3.21	0.57

**Table 2:** Correlation test of slice thickness and bleeding volume

Variable	*N*	*R*	*R* Square	Adjusted *R* Square	Std Error of the estimate	Significant *(p value)*
Slice thickness	68	0.79	0.62	0.61	12.72	0.00

Patients were categorized based on their gender and age. Patients were classified into 35 male patients (51.5%) and 33 female patients (48.5%). Table 3 shows statistical results of gender and age categories. The results of brain bleeding volume in male and female patients were (22.63 ± 3.74) ml and (20.84 ± 3.26) ml respectively. The results of brain bleeding volume in patients who ages lower than 50 years old and more or equal to 50 years old were (18.93 ± 3.26) ml and (23.53 ± 3.47) ml respectively. Brain bleeding volume were classified into 4 groups; Small (less than 10 ml), moderate (10-20 ml), large (21-30 ml), and very large (over 30 ml). The correlation between patient’s age and brain bleeding volume is not significant; *p* value is 0.18, with r value of 0.19, *N*= 68 as shown in Table 4.

**Table 3:** Statistical result and cross tabulation of patient’s age group and brain bleeding volume

Categories		*N*	Mean (ml)	*SD*	*SE*	Sample of Brain Bleeding Volume*			

						*S*	*M*	*L*	*VL*
Gender	Male	35	22.63	22.15	3.74	12	8	6	9
	Female	33	20.84	18.72	3.26	8	13	7	5
Age	(<50)	26	18.92	16.62	3.26	7	11	4	4
	(≥ 50)	42	23.53	22.47	3.47	13	10	9	10

**TOTAL**		**68**	**21.76**	**20.43**	**5.21**	**20**	**21**	**13**	**14**

* *S* = Small (<10 ml), *M* = Moderate (10-20 ml), *L*=Large (21-30 ml), *VL*=Very Large (>30 ml).

**Table 4:** Correlation test of patient’s age and brain bleeding volume

Variable	*R*	*R* Square	Adjusted *R* Square	Std Error of the estimate	Significant *(p value)*
Age	0.19	0.04	0.02	20.18	0.18

## 4. Discussion

Based on correlation analysis test result, a strong correlation existed between the number of slice thickness and brain bleeding volume with correlation coefficient of 0. 79 and linear regression test *p* value is 0.00. It shows that more slice thickness indicates a larger brain bleeding volume. The study result suggested that the dominant factor determining the size of brain bleeding volume on haemorrhagic stroke cases is the number of slices, with determinant factors of 62 %. This indicates that number of slices will match the size of brain’s bleeding volume. However, 38% were affected by other factors. The conclusion is; slice thickness from MSCT imaging is an accurate method to measure brain bleeding volume in haemorrhagic stroke cases.

From this study shows that male and female shares the same risk for stroke as shown in Table 3. From 68 haemorrhagic stroke cases, 35 cases occur in male patients (51.5%) and 33 cases occur in female patients (48.5%), there are no significant differences in gender (only 3%) for haemorrhagic stroke incidence. This study also calculate mean brain hemorrhage volume in male and female patient as shown in Table 3.

This study shows that there are no correlation between patient’s age and brain bleeding volume due to haemorrhagic stroke. From our descriptive analysis of 68 haemorrhagic stroke cases with different ages, the youngest patient is 19 years old with brain bleeding volume of 33.43 ml which can be categorized as large bleeding volume; the oldest patient is 80 years old with brain bleeding volume of only 6.75 ml. The mean value of brain bleeding volume does not significantly differ between younger and older patients. This means that large brain’s bleeding volume in haemorrhagic stroke cases did not only occur in elderly patients, but it might also occur in younger patients. However, haemorrhagic stroke does more frequently occur in older age groups as the risk of stroke incidence increased in older age group (over 40 years old).

In order to accurately measure the brain bleeding volume, the slice thickness or the number of slices from MSCT examination is the best way to indicate the volume of brain bleeding. The more slice thickness obtained from MSCT examinations indicates that the actual volume of brain haemorrhage is larger.

## 5. Conclusion

From all of the process in this study, starting from data collection, data analysis, and review of study results, we summed up the conclusion of this study as follows:

The number of slices obtained from MSCT examination has strong correlation to the actual brain bleeding volume, with correlation coefficient, *r* of 0.79. Therefore, the volume of brain haemorrhage can be accurately measured using computer software in MSCT device, the result of this measurement is considered to be accountable and reliable for decision making of medical actions.

Most of the hemorrhagic case in our study occurred in elderly patients. Thus, case management should be done carefully to avoid further deterioration of patient’s condition. Early recognition of an accurate brain hemorrhage volume could significantly help physicians to decide the proper medical action and case management which should be taken.

The average brain hemorrhage volume at various age group were nearly the same, there is no correlation between age and brain hemorrhage volume in 68 hemorrhagic stroke cases, with correlation coefficient, *R* of 0.19. Likewise, the average brain hemorrhage volume in male and female patients were also nearly the same, there is no significant difference in brain hemorrhage volume regarding gender group.
